# Deciphering comprehensive features of tumor microenvironment controlled by chromatin regulators to predict prognosis and guide therapies in uterine corpus endometrial carcinoma

**DOI:** 10.3389/fimmu.2023.1139126

**Published:** 2023-03-03

**Authors:** Qihui Wu, Ruotong Tian, Jiaxin Liu, Chunlin Ou, Yimin Li, Xiaodan Fu

**Affiliations:** ^1^ Department of Gynecology, Xiangya Hospital, Central South University, Changsha, China; ^2^ National Clinical Research Center for Geriatric Disorders, Xiangya Hospital, Changsha, China; ^3^ Department of Pharmacology, School of Basic Medical Sciences, Shanghai Medical College, Fudan University, Shanghai, China; ^4^ Department of Pathology, School of Basic Medical Sciences, Central South University, Changsha, China; ^5^ Department of Pathology, Xiangya Hospital, Central South University, Changsha, China; ^6^ Department of Pathology, Fudan University Shanghai Cancer Center, Shanghai, China; ^7^ Department of Oncology, Shanghai Medical College, Fudan University, Shanghai, China

**Keywords:** chromatin regulator, tumor immune microenvironment, DNA methylation, prognostic model, immunotherapy, uterine corpus endometrial carcinoma

## Abstract

**Background:**

Dysregulation of chromatin regulators (CRs) can perturb the tumor immune microenvironment, but the underlying mechanism remains unclear. We focused on uterine corpus endometrial carcinoma (UCEC) and used gene expression data from TCGA-UCEC to investigate this mechanism.

**Methods:**

We used weighted gene co-expression network analysis (WGCNA) and consensus clustering algorithm to classify UCEC patients into Cluster_L and Cluster_H. TME-associated CRs were identified using WGCNA and differential gene expression analysis. A CR risk score (CRRS) was constructed using univariate Cox and LASSO-Cox regression analyses. A nomogram was developed based on CRRS and clinicopathologic factors to predict patients' prognosis.

**Results:**

Lower CRRS was associated with lower grade, more benign molecular subtypes, and improved survival. Patients with low CRRS showed abundant immune infiltration, a higher mutation burden, fewer CNVs, and better response to immunotherapy. Moreover, low CRRS patients were more sensitive to 24 chemotherapeutic agents.

**Conclusion:**

A comprehensive assessment of CRRS could identify immune activation and improve the efficacy of UCEC treatments.

## 1 Introduction

With an estimated 417,000 new cases and 97,000 deaths in 2020, uterine corpus endometrial carcinoma (UCEC) is the sixth most common type of gynecological cancer worldwide (1). Over the past 30 years, the overall incidence has increased by 132%, although advances in medical devices and treatments have led to a 15% reduction in mortality rates over the same period (2). Most endometrial cancers can be cured by hysterectomy if detected early with postmenopausal bleeding, but those with advanced disease have a poor prognosis. The combination of neoadjuvant chemotherapy and interval cytoreductive surgery tends to result in less perioperative morbidity and higher survival rates in advanced stages (3). Unfortunately, not all patients benefit from these treatments.

Immunotherapy strategies have been made possible by advances in our understanding of the molecular biology of endometrial cancer. Immune checkpoint blockade (ICB) may be effective to some extent in the treatment of advanced and metastatic endometrial cancer. For patients with advanced or recurrent mismatch repair-deficient (MMRd) disease, pembrolizumab, a humanized monoclonal antibody targeting programmed cell death protein 1 (PD-1), has been approved by the US Food and Drug Administration (FDA), demonstrating a favorable safety profile and durable antitumor activity in this subset of patients (4). Although the results are promising, tolerability is a concern, with two-thirds of patients experiencing adverse events (5). There are many factors that influence the efficacy of immunotherapy, including the tumor microenvironment (TME) and genomic mutagenesis. To improve treatment success rates and reduce clinical stress and patient burden, new prognostic predictors are urgently needed.

The transduction of cellular signals is required for cell identity, differentiation, and stress response (6), with the majority of signals converging on chromatin. Over the past decade, significant progress has been made in the understanding of how factors that act on chromatin regulate transcription to coordinate the establishment of gene expression programs (7). Aberrant expression of chromatin regulators (CRs) has a major impact on immune responses. For example, HJURP has been associated with immune cell infiltration and immune checkpoint expression in hepatocellular carcinoma and clear cell renal cell carcinoma (8–10). HMGB3 was a member of a family of chromatin-binding proteins that can modify DNA structure to facilitate transcription factor binding (11, 12). Previous studies suggested that HMGB3 facilitates the immune escape of breast cancer cells (13). APOBEC3G inhibited HIV replication by mediating extensive deamination of a cytosine residue in the minus strands of the virus, allowing the virus to evade innate immunity (14). Furthermore, aberrant expression of CRs has been shown to be associated with outcomes in a variety of cancers (15). RAC3 was an understudied paralog of the canonical RAC1 GTPase and was implicated in tumor cell proliferation and invasion (16, 17).

Currently, although studies focusing on individual CR aberrations have been widely investigated, whether or how CRs orchestrate tumor cells and other components in the TME in UCEC is largely unknown. In our study, by WGCNA and consensus clustering algorithm, we identified two distinct CR clusters with different clinical outcomes and TME related pathways. Subsequently, we constructed a CR risk score (CRRS) using Univariate Cox and LASSO-Cox regression analyses. The CRRS can reflect the different clinicopathological parameters, clinical outcomes, immune status, genomic alterations, DNA methylation status of immune-related genes, and therapeutic response. The results of this study may provide a prognostic and therapeutic indicator for UCEC patients.

## 2 Materials and methods

### 2.1 Data collection and processing

The detailed workflow of this study is shown in **Figure S1**. The R package “TCGAbiolinks” was used to download RNA-seq data (TPM values), somatic mutations, and copy number variation (CNV) data from the TCGA dataset for UCEC patients (18). The TCGA-UCEC methylation profiling (HM450) datasets and pan-cancer data of TCGA were obtained from UCSC Xena (https://xenabrowser.net/datapage/). Clinicopathological data of UCEC patients were obtained from the cBioPortal (http://www.cbioportal.org/datasets). Patients with incomplete overall survival information were excluded from the study, which included 525 tumor tissues and 35 normal tissues. The term “entire cohort” refers to the total number of UCEC patients. Patients in the total cohort were then divided into two 1:1 cohorts, the training cohort and the validation cohort. The expression data of GSE17025 was downloaded from the Gene Expression Omnibus (GEO) (http://www.ncbi.nlm.nih.gov/geo/), consisting of 12 normal tissues and 91 tumor tissues. The immune checkpoint blockade treatment cohort (IMvigor210 cohort) was obtained from http://research-pub.Gene.com/imvigor210corebiologies. The R package “maftools” and the “ComplexHeatmap” were used to analyze and visualize the mutation data. GenePattern (https://www.genepattern.org/) was used to investigate the CNV of UCEC patients by GISTIC 2.0. A list of CRs was downloaded from a previously published article (19).

### 2.2 Weighted gene co-expression network analysis

We calculated the enrichment score of TME related pathways in TCGA-UCEC datasets using the ssGSEA method and the ESTIMATE algorithm (20, 21). Weighted gene co-expression network analysis (WGCNA) was then used *via* the R package “WGCNA” to better understand the relationships between CRs and the TME (22). A standard scale-free network and a topological overlap matrix (TOM), which is used to describe the similarity of gene expression to divide the genes with similar expression levels into different modules, were constructed using a soft threshold of β = 9 (scale-free R^2^ = 0.853). Candidate modules related to the TME were selected based on their high correlation coefficient. Gene significance (GS) was defined as the absolute value of the correlation between the gene and the TME. Gene significance (GS) was defined as the absolute value of the correlation coefficient between the gene expression and the estimated score of TME related pathways.

### 2.3 Human tissue specimens

A total of 24 endometrial carcinoma samples were collected from the Xiangya Hospital of Central South University.

### 2.4 RNA extraction and real-time PCR

Total RNA was obtained from cells using the FFPE RNA Extraction Kits (AmoyDx, Xiamen, China) in accordance with the manufacturer’s protocols. The quantity and quality of the extracted RNA was determined using a NanoDrop 1000 Spectrophotometer (Thermo Fisher, USA). The RNA samples were considered acceptable if they had an OD260/OD280 ratio within the range of 1.8-2.0 and an OD260/230 ratio within the range of 2.0-2.2. Following that, total RNA (1 μg) was used to inversely transcript the first-strand cDNA using HiScript II Reverse Transcriptase (Vazyme, Nanjing, China). Quantitative real-time PCR (qRT-PCR) was conducted on an ABI Prism 700 thermal cycler (Applied Biosystems, Foster City, CA, USA) as previously described (23). For RNA quantification, GAPDH was used as a normalizer. All experiments were performed in triplicate. Here are the primer sequences: APOBEC3G (forward primer: CCATCTTTGTTGCCCGCCTCTAC; reserve primer: GCAGGACCCAGGTGTCATTGTG); GAPDH (forward primer: AACGGATTTGGTCGTATTGG; reserve primer: TTGATTTTGGAGGGATCTCG).

### 2.5 Consensus clustering analysis

The single-sample gene set enrichment analysis (ssGSEA) algorithm was selected to evaluate the module scores. Unsupervised clustering analysis was applied to identify CR patterns based on the ssGSEA score of four modules and to classify patients for further analysis. The algorithm was performed using the “ConsensuClusterPlus” R package and the process was repeated 1,000 times to ensure the stability of the classification (24).

### 2.6 Construction and validation of a CR risk score

First, the correlations between the CRs and ImmuneScore or StromalScore were analyzed. A total of 369 genes were screened out with the threshold of |GS| > 0.3. Subsequently, the “limma” package was used to identify differentially expressed CRs between normal and tumor samples using the criteria of |log2 fold change (FC)| > 0.5 and an adjusted *p*-value < 0.05. Using GS and differential gene expression analysis, we discovered 86 TME-associated CRs. A cut-off *p*-value < 0.05 was used to screen TME-associated CRs with prognostic potential in the training cohort using a univariate Cox analysis of the overall survival (OS) in UCEC patients. The prognostic risk signatures of 9 TME-associated CRs were then determined in the training cohort using the Least Absolute Shrinkage and Selection Operator (LASSO) Cox regression analysis. The risk score (CRRS) was determined as follows:


$$CRRS=\sum_{i=1}^{n}\text{Coefi }{\mathrm{* x}}_{i}$$

(Coefi stands for coefficients, *x_i_* which are the expression levels of each prognostic gene.)

The CRRS of each patient in the training and validation cohorts was calculated separately using this formula. UCEC patients were then divided into low and high CRRS groups based on the median CRRS of the training cohort. The Kaplan-Meier method was used to compare the survival differences between the two CRRS groups, and the Log-rank test was used to determine statistical significance. Univariate and multivariate Cox regression analyses were used to confirm the prognostic value of the CRRS.

### 2.7 Construction of a predictive nomogram

Univariate and multivariate Cox regression confirmed that CRRS and stage were independent prognostic variables. Based on the CRRS and stage, a nomogram was developed using the “rms” R package. Each patient had an integrated score based on their stage and CRRS group. Using the integrated scores, we can predict the 1-/3-/5-year OS. Calibration curves and decision curve analysis (DCA) were used to demonstrate agreement between the practical outcome and the model prediction of outcome and clinical benefit.

### 2.8 Functional enrichment analysis

Enrichment analyses for Gene Ontology (GO) and the Kyoto Encyclopedia of Genes and Genomes (KEGG) (25) were performed using the R package “clusterProfiler”. The inclusion criteria were *p*-value < 0.05 and q-value < 0.05. For gene set variation analysis (GSVA) and gene set enrichment analysis (GSEA), the R packages “GSVA” and “clusterProfiler” were used to reveal the differences in biological functions and signaling pathways between the low and high CRRS groups (20, 25, 26). As the reference molecular signature dataset, the gene sets “h.all.v7.5.1”, “c2.cp.kegg.v7.5.1”, “c5.go.bp.v7.5.1”, and “c5.go.mf.v7.5.1” were retrieved from MSigDB (https://www.gsea-msigdb.org/gsea/msigdb/index.jsp). The top 10 significant pathways that were activated or inhibited (adjusted *p*-value < 0.05) were displayed.

### 2.9 Immune landscape analysis

As in our previous study, immune scores, stromal scores, estimate scores, and tumor purity of each sample were calculated using the ESTIMATE algorithm (21, 27). ssGSEA, CIBERSORT, IBERSORT-ABS, EPIC, TIMER, QUANTISEQ, MCPCOUNTER, and CXCELL were used to calculate the immune cell infiltration score (28–33). Wherein the immune cell marker in the ssGSEA algorithm was downloaded from the previous article (**Table S1**) (34). The TIP (http://biocc.hrbmu.edu.cn/TIP/index.jsp) was also used to download the cancer-immunity cycle of UCEC patients (35).

### 2.10 Immunotherapy/chemotherapy response

The Tumor Immune Dysfunction and Exclusion (TIDE) score, T cell exclusion level, and T cell dysfunction level were calculated using the TIDE algorithm (36). The Cancer Immunome Atlas (TCIA, https://tcia.at/home) provided the immunophenoscore (IPS) of UCEC patients (37). The stronger the immunogenicity, the higher the score. The submap analysis was used to predict anti-PD-1 and anti-cytotoxic T-lymphocyte-associated antigen-4 (CTLA-4) responses in patients with low- or high-CRRS (38). Based on the Genomics of Drug Sensitivity in Cancer (GDSC) (https://www.cancerrxgene.org/), the R package “pRRophetic” was used to predict the chemotherapy response of each sample (39). The relationship between drug sensitivity and the CRRS was investigated using a Spearman correlation analysis.

### 2.11 Statistical analysis

The continuous variables that were not normally distributed were compared using the Wilcoxon test (two groups) and the Kruskal-Wallis’s test (more than two groups). The chi-square (χ2) test was used to test categorical variables. The association between two continuous variables was calculated using Spearman’s correlation test. R software (version 4.0.5) was used to conduct all analyses. The following is how statistical significance was defined: ns stands for “not significant”, *p < 0.05, **p ≤ 0.01, ***p ≤ 0.001.

## 3 Results

### 3.1 Identification of endometrial carcinoma subtypes based on WGCNA and consensus clustering

**Figure 1:**
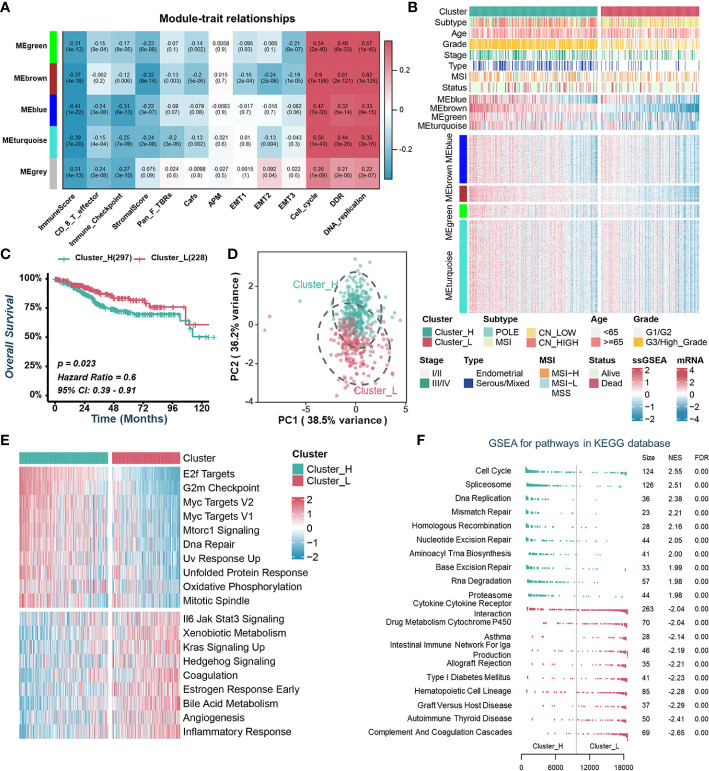
Identification of endometrial carcinoma subtypes based on WGCNA and consensus clustering. **(A)**. Heatmap of the correlation between module eigengenes with tumor microenvironment-related signatures or biological characteristics of tumor cells. **(B)**. Heatmap illustrating the expression pattern of CRs and the ssGSEA scores of modules between different clusters. **(C)**. Kaplan–Meier curve of OS between two TCGA-UCEC clusters. **(D)**. Principal component analysis to differentiate Cluster_H from Cluster_L. **(E)**. GSVA analysis of Hallmark gene sets in Cluster_H and Cluster_L. **(F)**. GSEA analysis of KEGG pathway gene sets in Cluster_H and Cluster_L.

Aberrant expression of chromatin regulators tends to dramatically remodel gene expression profiles dramatically. To investigate how CRs affect the TME of UCEC, we extracted the transcriptome data of 870 chromatin regulators from TCGA-UCEC dataset and performed WGCNA. β=9 was selected to construct a standard scale-free network using the Pick Soft Threshold function, and genes were subsequently assigned to four distinct modules using a cluster dendrogram (**Figures S2A, S2B**). In an effort to uncover potential modules that regulate the TME of UCEC, a correlation analysis was performed between each module eigengene and features assessing the TME or biological characteristics of malignant tumor cells. The result showed that MEgreen, MEblue, MEbrown and MEturquoise were negatively correlated with immune-related pathways while positively correlated with cell proliferation and tumor growth (**Figure 1A**). Specifically, the MEbrown was mainly positively correlated with the cell cycle, DNA damage response (DDR) and DNA replication, while negatively correlated with ImmuneScore and StromalScore (**Figure 1A**, **Figure S2C**). MEblue was negatively correlated with ImmuneScore, CD 8 T effector and immune checkpoint (**Figure 1A**, **Figure S2C**).

To explore the value of these modules in the prognosis of UCEC patients, we quantified the enrichment level of each module in each UCEC patient by using the ssGSEA method based on mRNA levels of genes from four modules, separately (**Figure 1B**). Kaplan-Meier analyses and univariate Cox regression analyses showed that patients with lower MEbrown had a more favorable OS than those with higher MEbrown scores (**Figures S2D, S2E**). Furthermore, MEbrown and MEturquoise scores were significantly positively correlated with cell proliferation and survival signatures, while negatively correlated with the ImmuneScore and StromalScore (**Figure S2F**). Using the consensus clustering algorithm, we divided TCGA-UCEC samples into two clusters based on the enrichment levels of four modules (**Figure 1B**, **Figures S2G, S2H**). We named the clusters with high ssGSEA scores “Cluster_H” and the clusters with low ssGSEA scores “Cluster_L” (**Figure 1B**, **Figure S2I**). Kaplan-Meier analyses showed that patients in Cluster_H had worse prognosis than those in Cluster_L (**Figure 1C**). Principal component analysis (PCA) displayed that the distributions of the two clusters were relatively scattered (**Figure 1D**). Furthermore, GSVA and GSEA analysis revealed that the cell proliferation and survival signatures were prominently enriched in the Cluster_H, whereas immune-related pathways were significantly enriched in the Cluster_L (**Figures 1E, F**).

### 3.2 Identifying immune-related CRs and constructing a risk score

To further investigate the relationship between CRs and TME, we examined the correlation between CRs and ImmuneScore or StromalScore, and screened out 369 genes with the threshold set as GS > 0.3. Subsequently, the mRNA expressions of these 369 CRs were compared between UCEC and normal tissues, of which 34 CRs were upregulated and 52 CRs were downregulated in UCEC samples (**Figure S3A**). After WGCNA and differential gene expression analysis, we identified 86 CRs, termed as TME-associated CRs. Furthermore, potential biological functions of TME-associated CRs were uncovered using GO and KEGG analyses. Covalent chromatin modification, histone modification, chromosomal region, condensed chromosome, histone binding, and transcriptional coregulator activity were found to be the common GO terms for these TME -associated CRs (**Figure S3B**). Furthermore, KEGG analysis revealed that these TME-associated CRs were enriched in functions such as lysine degradation, human immunodeficiency virus 1 infection, transcriptional misregulation in cancer and viral life cycle-HIV-1 (**Figure S3C**).

**Figure 2:**
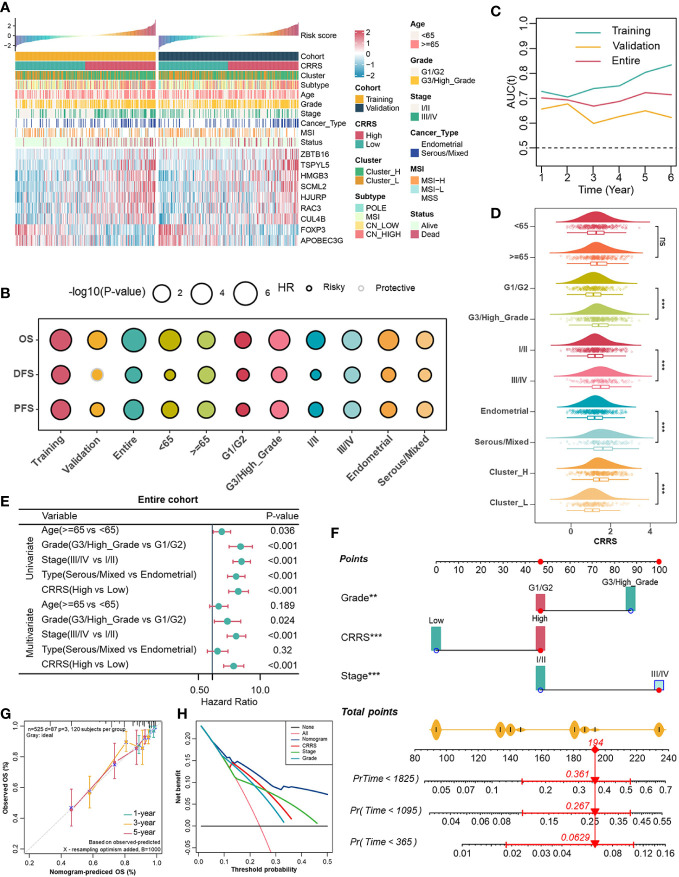
Associations between CRRS and clinicopathological features. **(A)**. Correlations between CRRS and 9 TME-associated CRs, clinicopathological features. **(B)**. Prognostic performance of the CRRS in different cohorts, age, grade, stage, and histological type. **(C)**. Time-dependent AUC value in the training, validation, and entire cohort. **(D)**. The distribution of CRRS among different clinicopathological features in the entire cohort. **(E)**. Univariate and multivariate Cox regression analyses evaluating independently predictive ability of CRRS and other clinicopathological features for OS in the entire cohort. **(F)**. Multivariate Cox regression analysis nomogram for predicting EC patients’ 1-/3-/5-years overall survival. **(G)**. Calibration curve for predicting OS at 1, 3, and 5 years. **(H)**. Decision curves for 5-year-OS in the entire cohort.

Subsequently, individuals from the entire cohort of TCGA-UCEC (n = 525) were randomly divided into the training cohort (n = 263) and the validation cohort (n = 262) to investigate the prognostic value of 86 TME-associated CRs. In the training cohort, using Univariate Cox and LASSO-Cox regression analyses, we constructed a CR risk score (CRRS) consisting of 9 TME-associated CRs: FOXP3, APOBEC3G, CUL4B, RAC3, HJURP, SCML2, HMGB3, TSPYL5, and ZBTB16 (**Figure 2A**, **Figures S3D, S3E**). The risk score formula was as follows: CRRS =-0.5456*FOXP3 - 0.2437*APOBEC3G + 0.0111*CUL4B + 0.0772*RAC3 + 0.1461*HJURP + 0.2032*SCML2 + 0.2631*HMGB3 + 0.2847*TSPYL5 + 0.4682*ZBTB16 (**Figure S3F**).

We then summarised the expression levels, the incidence of CNV, and somatic mutations of 9 genes were summarized in UCEC samples. In the TCGA-UCEC dataset, APOBEC3G, CUL4B, SCML2, TSPYL5, and ZBTB16 were significantly downregulated in tumor samples, whereas FOXP3, HJURP, HMGB3, and RAC3 were significantly upregulated in tumor samples (**Figure S4A**), which was generally consistent with the result observed in GSE17025 (**Figure S4B**). In addition, the relatively low mRNA levels of TSPYL5 and APOBEC3G in tumors might be related to hypermethylation of promoters (**Figure S4C**). Widespread CNV alterations might explain significantly upregulation of RAC3 in TCGA-UCEC (**Figure S4D**). CUL4B and SCML2 had relatively high mutation frequency (**Figure S4E**).

### 3.3 Investigating associations between the CRRS with clinicopathological parameters and improving the prognostic estimation system

In order to globally illustrate the relevance of CRRS in clinicopathological characteristics and its practical value for assessing the prognosis of UCEC patients, we first divided UCEC patients into low- and high-CRRS groups based on the median CRRS value in the training cohort, and this cut-off value was also applied in the entire and the validation cohorts (**Figure 2A**). Patients in the low-CRRS group were characterized by lower grade and stage, and relatively benign molecular genetic features, which was consistent with the survival advantage shown by the log-rank test (**Figures 2A, B**). Strikingly, patients with lower CRRS had an absolute prognostic advantage, whether in the training, validation, or entire cohort, even when more detailed clinicopathological characteristics including age, grade, stage, and histological types were taken into consideration (**Figure 2B**). The time-dependent AUC confirmed the predictive accuracy of the CRRS (**Figure 2C**). Previous studies have revealed that UCEC patients can be classified into four molecular subtypes based on the characteristics of their tumors, including POLE ultra-mutated, microsatellite instability hypermutated, copy-number low, and copy-number high (CN-high) (40). These subtypes have been shown to have prognostic significance, with the CN-high subtype being associated with a relatively poor prognosis among the four subtypes (40). In terms of clinicopathological characteristics, relatively high CRRS was observed in the higher grade and stage groups, in the serous/mixed histological types, in Cluster_H and in the copy number high (CN-high) molecular subtype (**Figure 2D**, **Figure S5A**). To further evaluate the predictive performance of CRRS in UCEC patients, we compared the CRRS with the Wang’s. sig and the Yao’s. sig (41, 42), and discovered that the AUC of OS for the CRRS is higher than that of other signatures (**Figure S5B**). We then examined the relationship between CRRS and OS in the TCGA pan-cancer dataset, and patients were divided into low- and high-CRRS groups based on the best cut-off value for each cancer type. The CRRS was identified as a risk factor in 11 cancer types (**Figure S5C**).

According to univariate and multivariate Cox regression analyses, the CRRS was an independent prognostic indicator for UCEC patients (**Figure 2E**, **Figures S5D, S5E**). To better assess the prognosis of UCEC patients, we constructed a nomogram to predict the 1-/3-/5-year survival probability in the entire cohort (**Figure 2F**). The nomogram included three independent prognostic factors including CRRS, grade, and stage (**Figure 2F**). There was excellent agreement between nomogram prediction and actual observation in the entire cohort at the 1-, 3-, and 5-year survival probabilities after calibration (**Figure 2G**). The net decision curve demonstrated the superiority of this nomogram in predicting the prognosis of UCEC patients (**Figure 2H**). The above results suggest that the CRRS, composed of TME-associated CRs, is indeed a prognostic indicator and is associated with clinicopathological features of UCEC.

### 3.4 Illustration of biological characteristics of different risk groups as determined by the appropriate CRRS value

**Figure 3:**
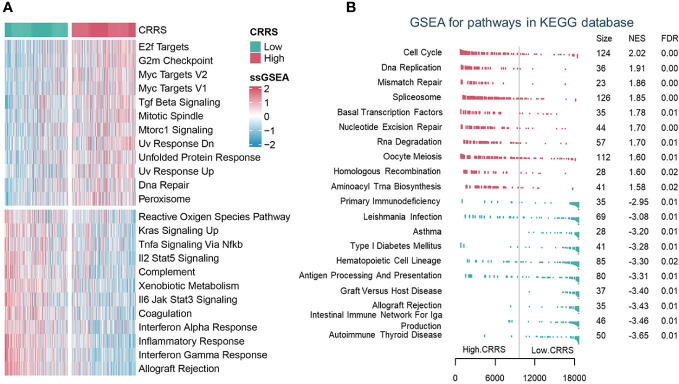
The biological characteristics of different risk groups. **(A)**. GSVA analysis of Hallmark gene sets in the low and high CRRS groups. **(B)**. GSEA analysis of KEGG pathway gene sets in groups with low and high CRRS.

To depict the biological characteristics of UCEC samples from different risk groups determined by the CRRS, we performed the GSVA and revealed that immune-related pathways such as allograft rejection, interferon-gamma response, inflammatory response, and interferon-alpha response were prominently enriched in the low-CRRS group, whereas E2F targets, G2M checkpoint, and Myc targets v1/v2 were markedly enriched in the high-CRRS group (**Figure 3A**). In addition, the GSEA was carried out with KEGG and GO gene set annotations. In the high-CRRS group, gene sets involved in cell proliferation and tumor growth were activated, but immune/inflammation-related pathways were suppressed (**Figures 3B**, **S6A, S6B**). Based on the concordance between the results of GSVA and GSEA, it was suggested that different immune response states might contribute to the different prognosis of UCEC patients in the two CRRS groups.

### 3.5 Uncovering the relevance of CRRS in the tumor immune microenvironment

**Figure 4:**
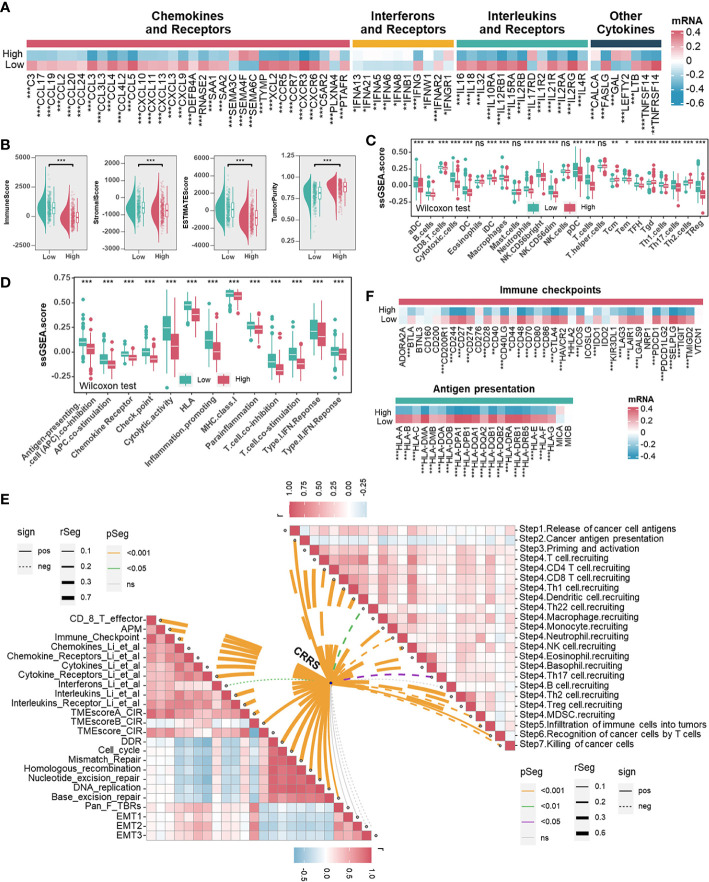
Associations between CRRS and the Tumor Microenvironment. **(A)**. Heatmap illustrating the expression pattern of chemokines, interferons, interleukins, other cytokines, and their receptors in different risk groups. **(B)**. The correlation between the CRRS and immune score, stromal score, ESTIMATE score, and tumor purity. **(C, D)**. TME infiltrating cell **(C)** and immune-related functions **(D)** comparisons between low- and high-CRRS groups. **(E)**. The lower left panel shows the correlations between the CRRS and immunoregulation-related pathways. The upper right panel shows the correlations between the CRRS and cancer immunity cycles. **(F)** Immune checkpoints and HLA family gene comparisons between low- and high-CRRS groups.

Immune/inflammation pathways were enriched or activated in UCEC samples from the low-CRRS group. However, the potential correlation between CRRS and the immune landscape of the UCEC remains unclear (**Figures 3A, B**, **S6A, S6B**). Cytokines (including but not limited to chemokines and interleukins) and their receptors were preferentially expressed higher in the low-CRRS group (**Figure 4A**). Using the ESTIMATE algorithm, we determined significantly higher immune, stromal, and estimate scores while lower tumor purity in the low-CRRS group (**Figure 4B**). By evaluating the infiltration of immune cells in UCEC samples with ssGSEA, we revealed that the low-CRRS group had more enrichment of cytotoxic CD8^+^ T cells, dendritic cells (DC), and natural killer (NK) cells (**Figure 4C**), which was confirmed by other independent algorithms (**Figure S7A**). The relationship between nine TME-associated CRS and CD8^+^ T cells was further confirmed by a variety of algorithms, and the outcome demonstrated that CRs were connected to CD8^+^ T cells (**Figure S8A**). Among them, APOBEC3G was found to be positively correlated with CD8^+^ T cells and the CD8^+^ T effector signature, and its high expression was associated with the prognosis of patients (**Figures S8A–S8C**). We further verified the relationship between APOBEC3G and CD8A/GZMB by qRT-PCR, and the results demonstrated that the two variables were positively correlated (**Figure S8D**). Then, we investigated the heterogeneity expression pattern of APOBEC3G, CD8A, and GZMB in different immune cells at a single-cell level using TISCH2 (UCEC-GSE139555) (43), and found that they were mainly expressed in CD8^+^ T cells (**Figure S8E**). Next, we explored associations between CRRS and signatures of antitumor immunity. Relatively activated antitumor functions of immune cells were revealed in the low-CRRS group (**Figure 4D**), and CRRS was negatively correlated with most immune-related functions as well as cancer immunity cycles (**Figure 4E**), which was consistent with immune activation status and better prognosis of UCEC patients with relatively low CRRS. In addition, significant upregulation of immune checkpoint genes and HLA family genes in the low CRRS group may indicate higher immune cell infiltration and more potentially presented neo-antigens (**Figure 4F**). Furthermore, CRRS was found to be negatively correlated with immune score, immune checkpoint, immune cells and immune-related pathways, and positively correlated with tumor purity and tumor growth-related pathways in the majority of cancers (**Figures S9A, S9B**).

### 3.6 Comprehensive genomic alterations analyses in different CRRS groups

**Figure 5:**
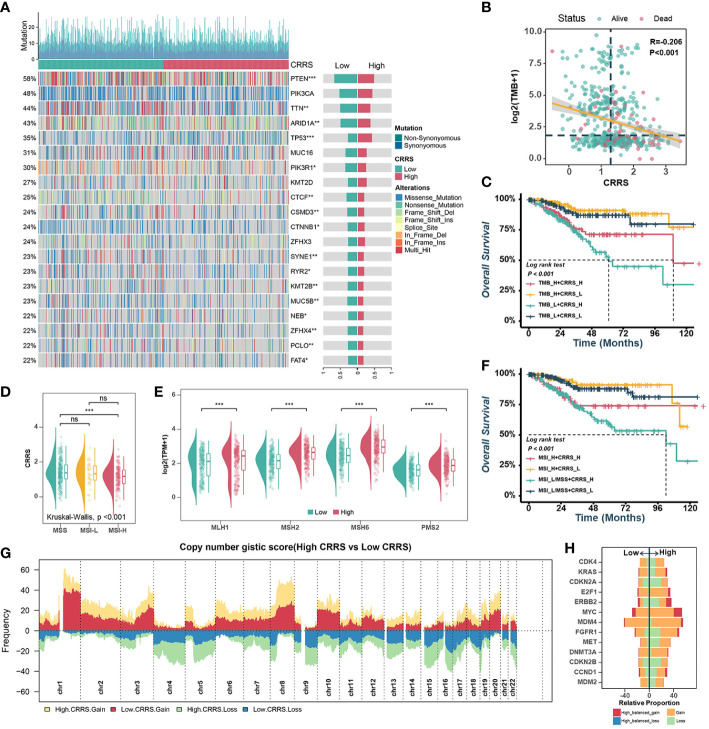
Comprehensive analyses of genomic alterations in different risk groups. **(A)**. Oncoplots of the top 20 most frequently mutated genes in the TCGA-UCEC dataset. Each column represents an individual patient. The small figure above shows the non-synonymous and synonymous mutation counts (log2). The figure on the right shows the mutation rates of different groups. **(B)**. Scatter plots showing the correlation between the CRRS and TMB. The color indicates different survival statues. **(C)**. Survival analyses for subgroup patients stratified by both CRRS and TMB. **(D)**. The prevalence of CRRS in the MSS, MSI-L, and MSI-H groups. **(E)**. Comparisons of MLH1, MSH2, MSH6, and PMS2 between low- and high-CRRS groups. **(F)**. Survival analyses for the subgroup of patients stratified by both CRRS and MSI. **(G)**. Gain and loss frequency in the low- and high-CRRS groups. **(H)**. The CNV of some representative oncogenes and tumor suppressors.

Previous research had supported that the response to ICB is closely associated with somatic mutation that increases tumor-specific neoantigens (40, 41). Firstly, the waterfall plot displayed the top 20 genes with the highest mutation rates in TCGA-UCEC datasets (**Figure 5A**). The gene with the highest mutation frequency was PTEN (58%), followed by PIK3CA (48%) and TTN (44%). When comparing the mutations in these genes between the different groups, it was found that the low-CRRS group presented more extensive somatic mutation, on the whole, than the high-CRRS group. Specifically, most genes such as PTEN, TTN and ARID1A were more frequently mutated in the low-CRRS group, whereas TP53 had a higher somatic mutation rate in the high-CRRS group (**Figure 5A**). Additionally, the CRRS was significantly inversely related to tumor mutation burden (TMB) (**Figure 5B**), and the combination of CRRS and TMB could better predict the overall survival of UCEC patients (**Figure 5C**). Microsatellite instability hypermutated (MSI-H) tumors account for approximately 25% to 30% of endometrial carcinomas (42), which have DNA mismatch repair defects, resulting in errors in repetitive DNA sequences known as microsatellites (41, 43). As shown in **Figure 5D**, the MSI-H subtype had a lower CRRS. Meanwhile, in the low-CRRS group, the expressions of MLH1, MSH2, MSH6, and PMS2 were significantly lower (**Figure 5E**). The UCEC patients with MSI-L/MSS and high CRRS had the worst prognosis, as shown by the Kaplan-Meier curves (**Figure 5F**). Previous studies (44) suggested that CNVs play an important role in tumorigenesis. Resistance to anti-CTLA-4 and anti-PD-1 blockade has been associated with a higher burden of copy number loss (44). In our study, high-frequency amplification or loss was discovered in the high-CRRS group (**Figure 5G**). Some of them are shown in **Figure 5H**. For example, recurrent amplification of oncogenes such as MYC, ERBB2 (HER2), and FGFR1, and significant loss of tumor suppressors such as CDKN2A and CDKN2B were observed in the high-CRRS group.

### 3.7 Investigating connections between the CRRS and DNA methylation of immune-related genes

**Figure 6:**
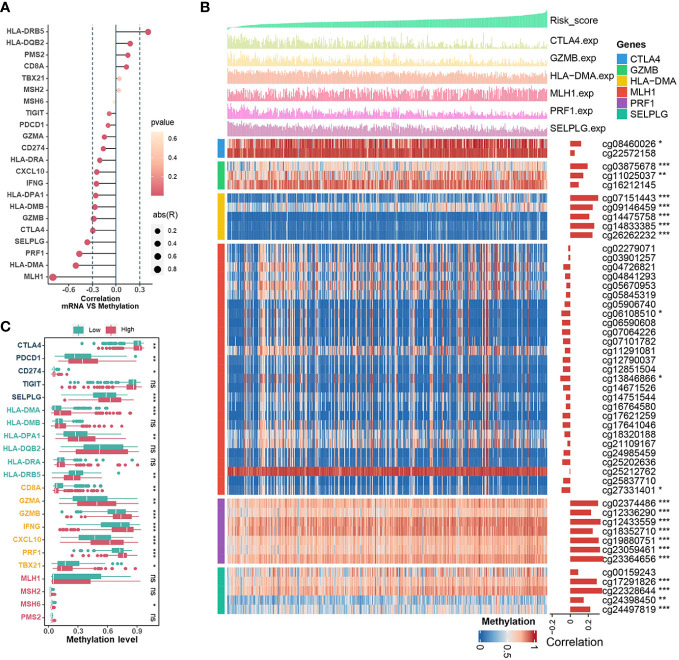
The Relationships Between CRRS and DNA methylation. **(A)**. The bubble chart shows the correlation between expression levels and methylation levels of the immune-related genes. The length of the vertical line indicates the degree of correlation, and the color indicates the *p*-value. **(B)** The correlation between the CRRS and the methylation levels of CpG sites in the promoter region of the MLH1, HLA-DMA, PRF1, SELPLG, CTLA4, and GZMB genes. **(C)**. Comparisons of the methylation levels of immune-relation pathway genes (Mismatch Repair signature, CD8^+^ T effector signature, Antigen presentation signature, and Immune Checkpoint signature) in low- and high-CRRS groups.

Essentially, CRs regulate events during gene transcription, where DNA methylation in the promoter region strongly influences the dysregulation of gene expression during tumor development. Therefore, we investigated the associations between CRRS and methylation levels in the promoters of genes involved in signatures such as mismatch repair (MLH1, MSH2, MSH6, PMS2), CD8^+^ T effector (CD8A, GZMA, GZMB, IFNG, CXCL10, PRF1, TBX21), antigen presentation (HLA-DMA, HLA-DMB, HLA-DPA1, HLA-DQB2, HLA-DRA, HLA-DRB5), and immune checkpoint (CTLA4, PDCD1, CD274, TIGIT, SELPLG). Among them, the expression levels of MLH1, HLA-DMA, PRF1, SELPLG, CTLA4 and GZMB in TCGA-UCEC were inversely correlated with their methylation levels (**Figure 6A**). The estimated CRRS scores showed a negative correlation with the methylation level of CpG sites within the promoter regions of HLA-DMA, PRF1, SELPLG, CTLA4, and GZMB (**Figure 6B**), and the methylation levels of these genes were significantly higher in the high-CRRS group (**Figure 6C**). Although PDCD1 expression was weakly negatively correlated with its methylation level (R = -0.10, p = 0.042), the methylation levels of PDCD1 were higher in the high-CRRS group (**Figures 6A, C**).

### 3.8 The role of the CRRS in the prediction of immunotherapy benefits and the selection of sensitive chemotherapeutic agents

**Figure 7:**
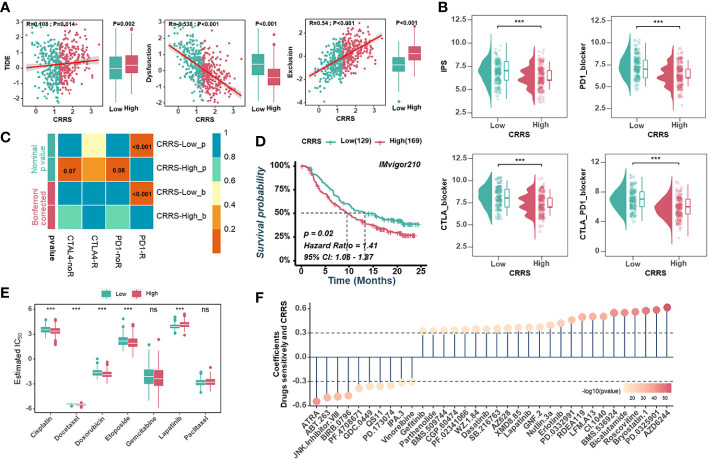
Predictive value of CRRS in immunotherapy and chemotherapy. **(A)**. Scatter plots(left) and box diagrams (right) show the correlations between the CRRS and the TIDE score, dysfunction score, and exclusion score. **(B)**. The relationship between the CRRS and IPS. **(C)**. The submap analysis predicts the probability of anti-PD1 and anti-CTLA4 immunotherapy response in low- and high-CRRS groups in the entire cohort. **(D)**. Kaplan-Meier curves for the IMvigor210 cohort’s low- and high-CRRS groups. **(E)**. Chemotherapeutic sensitivity of 7 common drugs (Cisplatin, Docetaxel, Doxorubicin, Etoposide, Gemcitabine, Lapatinib, and paclitaxel) was estimated and compared. **(F)**. The bubble chart shows the correlation between CRRS and drug sensitivity. The length of the vertical line indicates the CRRS related to drug resistance (R > 0.3) or drug sensitivity (R < -0.3) to the CRRS.

The high neoantigen load and immune activation implied that ICB might be effective and beneficial to the treatment of UCEC patients with relatively low CRRS. Firstly, we used the TIDE algorithm to assess the value of CRRS in predicting the potential clinical efficacy of immunotherapy. According to **Figure 7A**, the low-CRRS group had a higher dysfunction score, a lower TIDE score, and a lower exclusion score. Next, IPS was applied to assess the immunogenicity of UCEC samples, and the low-CRRS group had higher IPS, PD1-blocker, CTLA-blocker, and CTLA4-PD1-blocker scores (**Figure 7B**). We also used SubMap algorithms to investigate the response to immunotherapy targeting CTLA-4 and PD-1 in the low and high CRRS groups. We found that patients with low CRRS showed promising responses to anti-PD-1 therapy (**Figure 7C**). Given the high correlation between CRRS and immune response in UCEC, we further investigated whether CRRS could predict patients’ response to ICI therapy in an independent immunotherapy cohort. In the IMvigor210 cohort, the patients with high CRRS indeed had a worse prognosis (**Figure 7D**). These findings suggested that patients with low CRRS might have a better response to ICB therapy. Meanwhile, we also analyzed correlations between the CRRS and IC50 of drug candidates in the GDSC dataset. The sensitivity of seven commonly used chemotherapy drugs, including cisplatin, docetaxel, doxorubicin, etoposide, gemcitabine, lapatinib and paclitaxel, was investigated, and the estimated IC50 of cisplatin, docetaxel, doxorubicin, etoposide and lapatinib was found to be significantly different between the two groups (**Figure 7E**). Notably, the estimated IC50 of the 10 agents showed negative correlations with CRRS, meaning that these agents might benefit patients with high CRRS, while the other 24 drugs showed the opposite (**Figure 7F**). Taken together, these results suggested that the CRRS may be a promising biomarker for guiding precision treatment strategies.

## 4 Discussion

The TME not only plays a major role in tumor progression, but also orchestrates immune components, thus affecting the therapeutic efficacy of ICB and patient prognosis (44, 45). Recent research suggests that epigenetic changes, usually caused by chronic inflammation, occur in cancer cells and other TME components (46, 47). These changes can influence and modulate a variety of aspects of cancer progression, including tumor growth, metabolic state, metastatic spread, immune escape, and the generation of immunosuppressive or immunosupportive leukocytes (48). CRs have been identified as critical elements consisting of chromatin remodelers, DNA methylators and histone modifiers involved in epigenetic regulation (19, 49, 50). Whether and how these CRs manipulate the epigenetic variation of immune cells and shape the unique TME of UCEC is currently unknown. If this is the case, the therapeutic potential of immunotherapy such as ICB in combination with chemotherapeutic agents determined by CRs will be justified, considering that the combination of multiple therapeutic agents has been shown to be a successful strategy in oncology (7). Therefore, a comprehensive and in-depth study of CRs and the relationship between CRs and the immune microenvironment is required to identify patients who may benefit from new therapeutic strategies.

In this study, we collected transcriptome files of CRs from the TCGA-UCEC cohort and identified two subtypes based on WGCNA and consensus clustering. Tumor proliferation and survival signatures were prominently enriched in Cluster_H with a worse prognosis, whereas immune-related pathways were markedly enriched in Cluster_L with longer survival. Univariate Cox regression analyses and Kaplan-Meier analyses showed that MEbrown was correlated with prognosis. The results were particularly striking in the Cluster_H and Cluster_L groups, suggesting that Cluster_H is primarily driven by MEbrown. Meanwhile, we identified 86 TME-associated CRs by | GS | >0.3 and differential gene expression analyses, and created an immune-related CRRS based on the expression of 9 genes (FOXP3, APOBEC3G, CUL4B, RAC3, HJURP, SCML2, HMGB3, TSPYL5, and ZBTB16) through univariate cox regression and LASSO cox analyses. Since the risk score was determined as an independent prognostic factor for UCEC patients, a nomogram based on the CRRS and traditional clinicopathological characteristics was constructed to predict the 1-/3-/5-year survival possibility. To further explore the relationship between CRRS and cancer prognosis, a pan-cancer study was conducted using data from TCGA. Results of the study revealed that CRRS is a risk factor for 11 types of cancer, including cervical squamous cell carcinoma, endocervical adenocarcinoma, ovarian serous cystadenocarcinoma, breast invasive carcinoma, colon adenocarcinoma, stomach adenocarcinoma, and bladder urothelial carcinoma.

In general, 9 TME-associated CR genes could be categorized according to their essential functions. APOBEC3G is a DNA methylator and CUL4B, HJURP and ZBTB16 are histone modifiers, whereas the classification of FOXP3, HMGB3, RAC3, SCML2 and TSPYL5 is still unknown (19). RAC3 was elevated in EC patients and was associated with poor clinical outcome. A negative correlation was observed between the expression of RAC3 and the infiltration levels of B cells, CD8+ T cells, macrophages, and dendritic cells in EC (51). The RAC3 gene was amplified in breast cancer and correlated with tumor size and estrogen as well as progesterone receptor positivity (52). In our study, RAC3 was significantly upregulated in tumor samples and exhibited widespread CNV alterations. HMGB3 was found to be overexpressed in a variety of cancers, including breast invasive carcinoma, sarcoma, skin cutaneous melanoma, ovarian serous cystadenocarcinoma, and acute myeloid leukemia (53, 54). The upregulation of the HMGB3 gene has been implicated in tumorigenesis and chemotherapy resistance *via* various mechanisms (54). In UCEC, suppression of HMGB3 expression has been shown to impede the proliferation, migration, and invasion of EC cell lines (55).

After the CRRS was constructed and verified, TCGA-UCEC patients were divided into low - and high-CRRS groups according to the median value, with the high-CRRS group having worse clinical outcomes. Further analysis using GSVA and GSEA revealed that pathways associated with cell proliferation and tumor growth were activated in the high-CRRS group, whereas pathways related to immune/inflammation were enriched in the low-CRRS group. These results suggest that distinct immune response states may underlie the varying prognoses of UCEC patients. The relationship between CRRS and TME was further explored. Firstly, CRRS was inversely associated with ImmuneScore, StromalScore and EstimateScore, whereas it was positively associated with TumourPurity. We also investigated the relationships between CRRS and immune components and processes in the TME of UCEC. Recent studies suggest that chemokines can directly alter the tumor microenvironment to promote tumor growth by regulating pro-inflammatory signaling, immune cell infiltration and tumor metastasis (56). In our data, the samples from the low-CRRS group had higher levels of CCL20, CCR5, CCR7, CXCL10, CXCL11, and CXCR3, which are responsible for attracting DCs and CD8^+^ T cells (57, 58), supported by the finding that CRRS was negatively correlated with infiltration of antitumor immune cells, including CD8^+^ T cells, DC cells, and NK cells. Also, CRRS was generally negatively correlated with cancer immunity cycles, immune-related functions, and signatures associated with antitumor immunity. These findings point to a noninflamed phenotype of the high-CRRS group, implying a poor response to ICB therapy. In addition, the CRRS was found to have negative correlations with the immune score, immune checkpoint, immune cells, and immune-related pathways, while positive correlations with tumor purity and tumor growth-related pathways in most cancers. Recently, epigenetic reprogramming of exhausted CD8^+^ T cells has been identified as a limiting factor in long-term effective PD-1 blocker treatment (7, 59, 60). In particular, the role of DNA methylation in the regulation of PD-1 expression after T-cell receptor stimulation in an *in vivo* model of acute infection has been demonstrated (61, 62). When compared with normal tissues, CpG islands in the promoter regions of PD-1, CTLA-4, and TIM-3 were significantly hypomethylated in breast cancer (63). Methylation levels of the CD8^+^ T effector signature (GZMA, GZMB, IFNG, CXCL10, PRF1) and PD-1 were significantly lower in the low-CRRS group, according to our study.

There are four TCGA molecular subtypes of endometrial carcinomas: POLE mutated, MSI-H, copy-number low, and copy-number high (40). POLE-mutated and MSI-H endometrial cancers are linked to a high abundance of tumor-infiltrating lymphocytes and neoantigen loads, implying a more effective outcome with immunotherapy (64–66). MSI-H tumors have DNA MMRd, and MMR can occur sporadically as a result of methylation of the MLH1 promoter or germline mutations in MMR genes, as shown in Lynch syndrome (67). According to our study, there were significant negative correlations between CRRS and TMB, and MMR genes were expressed at a lower level (MLH1, MSH2, MSH6, and PMS2) in the low-CRRS group. Generally, TMB could prompt the production of mutation-derived neoantigens and thus enhance tumor immunogenicity, which further leads to the activation of cytotoxic T lymphocytes (68). In this study, we found that TMB was significantly higher in the low CRRS group than in the high CRRS group. In addition, immune-related CRRS showed better predictive performance when combined with TMB/MSI. Recent research has suggested that tumors with high CNV levels have a more severe tumorigenic and immunosuppressive immune microenvironment than tumors with low CNV levels (69). In this case, high frequency gain or loss was detected in the high CRRS group.

In addition, the TIDE, IPS, and SubMap algorithms have been used to predict patient response to ICB (36–38). The TIDE algorithm integrates the expression signatures associated with two major mechanisms of tumor immune evasion, namely T cell dysfunction and T cell exclusion, to evaluate tumor immune evasion and predict responsiveness to ICB therapy (36). This approach was designed to provide a more accurate biomarker for ICB response compared to traditional biomarkers (36). A higher TIDE score indicates a higher likelihood of tumor cells inducing immune evasion and a lower response rate to ICB treatment (70). In line with expectations, the low-CRRS group exhibited a significantly lower TIDE score, T cell exclusion score, and a higher T cell dysfunction score. The IPSs of UCEC patients were downloaded from the TCIA dataset, which can predict the response to CTLA-4 and PD-1 blockers (37). Higher scores were associated with better outcomes with ICB treatment (71). The low-CRRS group had a higher IPS, indicating a better response to immunotherapy. The SubMap algorithm was used to identify similarities in the expression profiles between TCGA-UCEC and melanoma patients treated with ICB (38, 72). This also confirmed that the low-CRRS group may respond better to ICB treatment. All these findings suggest that CRRS can be used as a promising predictor of response to immunotherapy in UCEC, which was validated in the IMvigor210 dataset. Notably, patients with a high CRRS did indeed have a worse prognosis.

Our research still has some limitations. For starters, as this study is primarily based on the TCGA dataset, which has a limited number of samples, it requires additional external datasets, in particular the immunotherapy chip for UCEC verification. Secondly, only GSE17025 from the GEO datasets was used to verify the results. Third, our research is based on bioinformatic analysis of data from public datasets. Using WGCNA and differential gene expression analysis, we discovered 86 CRs associated with ImmuneScore/StromalScore, which we termed TME-related CRs. However, further research is required to fully understand the specific functions and associated mechanisms of these genes, and additional clinical studies are needed to validate the accuracy of our model. Finally, CRs may have different functions in the TME of different tumors, and the immune-related CRRS we developed is primarily used to predict immunotherapy in UCEC.

## 5 Conclusions

This comprehensive and in-depth study helps to elucidate the role of chromatin regulators in the TME of UCEC. Two distinct CR clusters were identified that were associated with different clinical outcomes and biological characteristics. Meanwhile, we developed a risk score based on CRs that predicts the prognosis of UCEC. Using this prognostic score, we evaluated clinical outcomes, biological characteristics, immune status, and genomic alterations in different CRRS groups. The predictive value of CRRS in immunotherapy and chemotherapy suggests that CRRS may be a promising biomarker for the development of precision treatment strategies for UCEC.

## Data availability statement

The original contributions presented in the study are included in the article/**Supplementary Material**. Further inquiries can be directed to the corresponding authors.

## Author contributions

Conceptualization, YL, QW and RT; formal analysis, YL; funding acquisition, CO; investigation, QW; methodology, XF; project administration and supervision, CO, XF and YL; visualization, JL; writing—original draft, RT; writing—review and editing, QW. All authors contributed to the article and approved the submitted version.

## Supplementary material

Supplementary Figure 1 Flow chart of this study.

Supplementary Figure 2 Identification of endometrial carcinoma subtypes based on WGCNA and consensus clustering. **(A)**. Scale-independence analysis(left) and mean connectivity analysis (right) for various soft-thresholding power values. **(B)**. Dendrogram of all chromatin regulators clustered based on a dissimilarity measure (1-TOM). **(C)**. Module eigengene scatter plots in the brown and blue modules. **(D, E)**. Kaplan–Meier curves **(D)** and Univariate Cox regression analyses **(E)** of OS between the different groups based on the ssGSEA scores of modules. **(F)**. The correlation between the ssGSEA scores of modules with tumor microenvironment-related signatures or biological characteristics of tumor cells. **(G, H)**. CDF plot and Consensus matrices of TCGA-UCEC for k = 2. **(I)**. The ssGSEA scores of modules comparisons between Cluster_H and Cluster_L

Supplementary Figure 3 Establishment of the immune-related chromatin regulator prognostic signature. **(A)**. Volcano plot showed differentially expressed 86 CRs in UCEC compared with normal tissues. **(B, C)**. Gene Ontology analysis **(B)** and KEGG pathway enrichment analysis **(C)** for immune-related CRs. **(D)**. The LASSO coefficient profile of TME-associated CRs was drawn *via* 10-fold cross-validation. **(E)**. The tuning parameters (log λ) of TME-associated CRs were selected to cross-verify the error curve. **(F)**. Coefficients of 9 TME-associated CRs were finally obtained in the prognostic signature.

Supplementary Figure 4 The expression and genetic alterations of 9 TME-associated CRs in endometrial carcinoma. **(A, B)**. The difference in mRNA expression levels of 9 TME-associated CRs between normal and endometrial carcinoma samples in TCGA-UCEC (A) and GSE17025 **(B)**. **(C)**. The correlation between expression levels and methylation of promoters of 9 TME-associated CRs. **(D, E)**. The CNV frequency **(D)** and the mutation frequency **(E)** of 9 TME-associated CRs were prevalent.

Supplementary Figure 5 Associations between CRRS and clinicopathological features. **(A)**. The distribution of CRRS in TCGA-UCEC molecular subtypes in the entire cohort. **(B)**. The AUC for CRRS and other prognostic signatures in the entire cohort. **(C)**. Prognostic performance of the CRRS in the TCGA pan-cancer dataset. **(D, E)**. Univariate and multivariate Cox regression analyses were performed in the training **(D)** and validation **(E)** cohorts to assess the independent predictive ability of CRRS and other clinicopathological features for OS.

Supplementary Figure 6 The biological characteristics of different risk groups. **(A)**. GSEA analysis of biological process gene sets from the GO dataset. **(B)**. GSEA analysis of molecular function gene sets from the GO dataset.

Supplementary Figure 7 Relationships between CRRS and the Tumor Microenvironment. **(A)**. Relative cell abundance of CD8^+^ T cells, macrophages, DC cells, and NK cells were calculated by different algorithms in the low- and high-CRRS groups.

Supplementary Figure 8 APOBEC3G was positively correlated with CD8^+^ T cells in UCEC. **(A)**. Multivariate analysis confirmed the correlation between the 9 TME-associated CRs and the levels of infiltration of CD8^+^ T cells. **(B)** Kaplan–Meier curve for OS between high- and low- APOBEC3G groups. **(C)** Correlation between the APOBEC3G and CD8^+^ T effector signature in TCGA-UCEC. **(D)** Validation of the correlation between APOBEC3G and CD8/GZMB by qRT-PCR. **(E)** The results of APOBEC3G, CD8, GZMB expression distribution in single cell dataset (UCEC-GSE139555).

Supplementary Figure 9 CRRS and Tumor Microenvironment associations in the TCGA pan-cancer dataset. **(A)**. The correlation between the CRRS and immune score, stromal score, ESTIMATE score, and tumor purity. **(B)**. Heatmap illustrating the correlation between the CRRS and immune checkpoints, immune cells, and tumor microenvironment-related signatures.
