# Supplementary figures and images for: Deciphering comprehensive features of tumor microenvironment controlled by chromatin regulators to predict prognosis and guide therapies in uterine corpus endometrial carcinoma
Source: Front Immunol. 2023 Mar 3;14:1139126. doi: 10.3389/fimmu.2023.1139126 (PMC10022674; doi:10.3389/fimmu.2023.1139126)

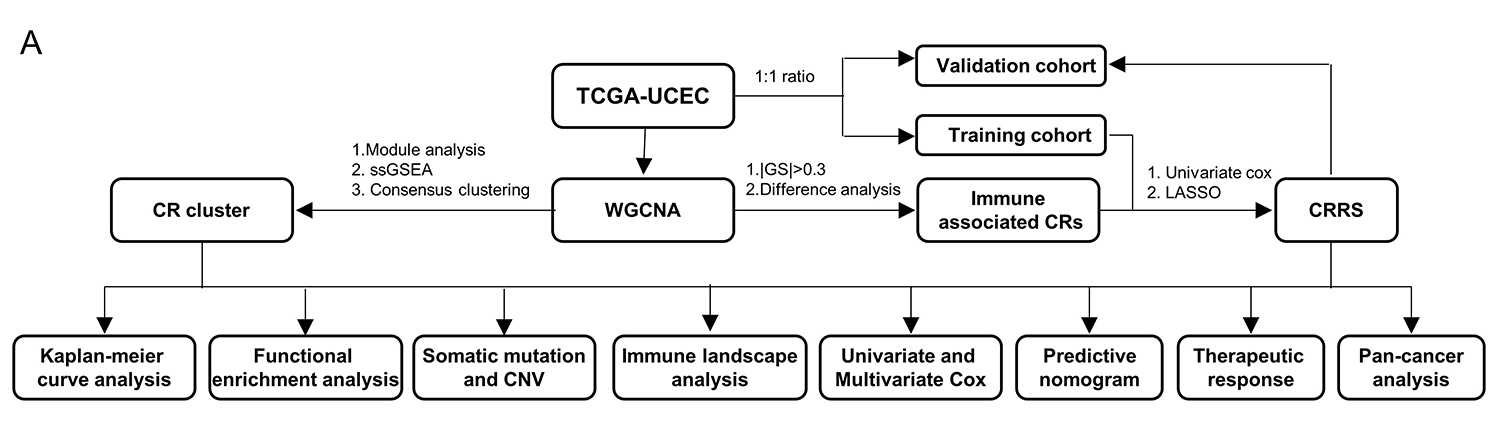

Supplement: Supplementary Figure 1 — Flow chart of this study. [file Image_1.jpeg]

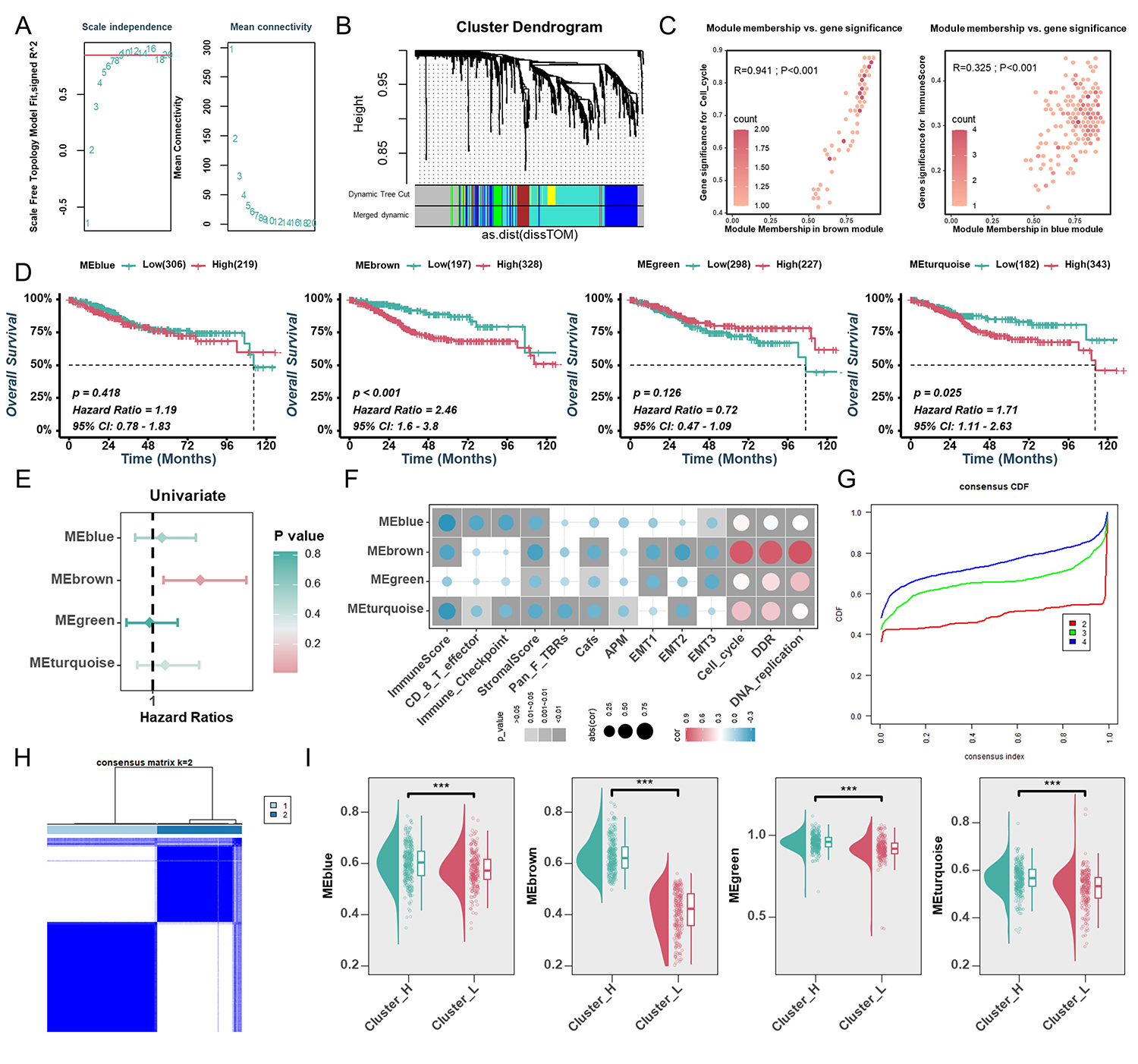

Supplement: Supplementary Figure 2 — Identification of endometrial carcinoma subtypes based on WGCNA and consensus clustering. (A). Scale-independence analysis(left) and mean connectivity analysis (right) for various soft-thresholding power values. (B). Dendrogram of all chromatin regulators clustered based on a dissimilarity measure (1-TOM). (C). Module eigengene scatter plots in the brown and blue modules. (D, E). Kaplan–Meier curves (D) and Univariate Cox regression analyses (E) of OS between the different groups based on the ssGSEA scores of modules. (F). The correlation between the ssGSEA scores of modules with tumor microenvironment-related signatures or biological characteristics of tumor cells. (G, H). CDF plot and Consensus matrices of TCGA-UCEC for k = 2. (I). The ssGSEA scores of modules comparisons between Cluster_H and Cluster_L [file Image_2.jpeg]

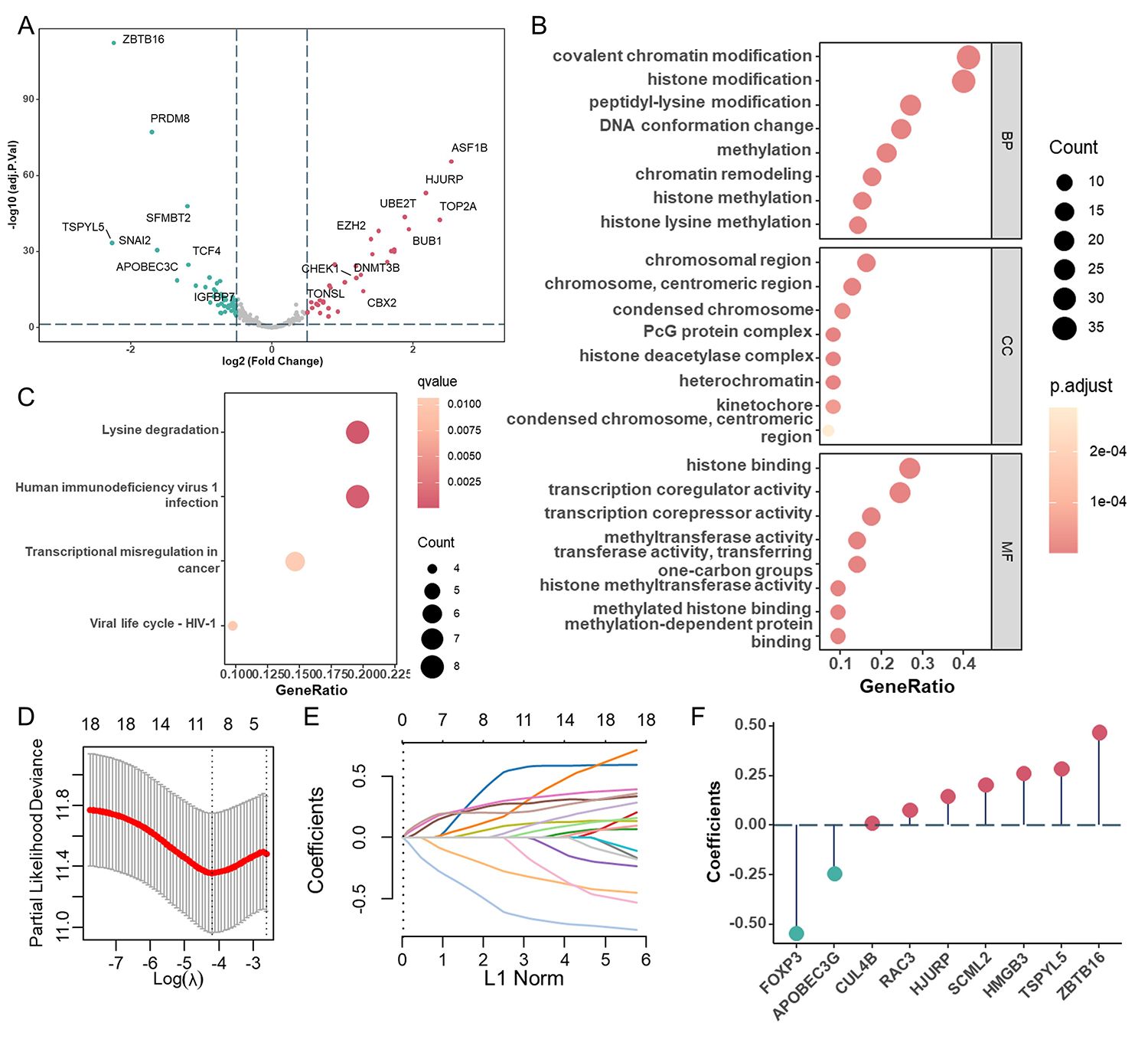

Supplement: Supplementary Figure 3 — Establishment of the immune-related chromatin regulator prognostic signature. (A). Volcano plot showed differentially expressed 86 CRs in UCEC compared with normal tissues. (B, C). Gene Ontology analysis (B) and KEGG pathway enrichment analysis (C) for immune-related CRs. (D). The LASSO coefficient profile of TME-associated CRs was drawn via 10-fold cross-validation. (E). The tuning parameters (log λ) of TME-associated CRs were selected to cross-verify the error curve. (F). Coefficients of 9 TME-associated CRs were finally obtained in the prognostic signature. [file Image_3.jpeg]

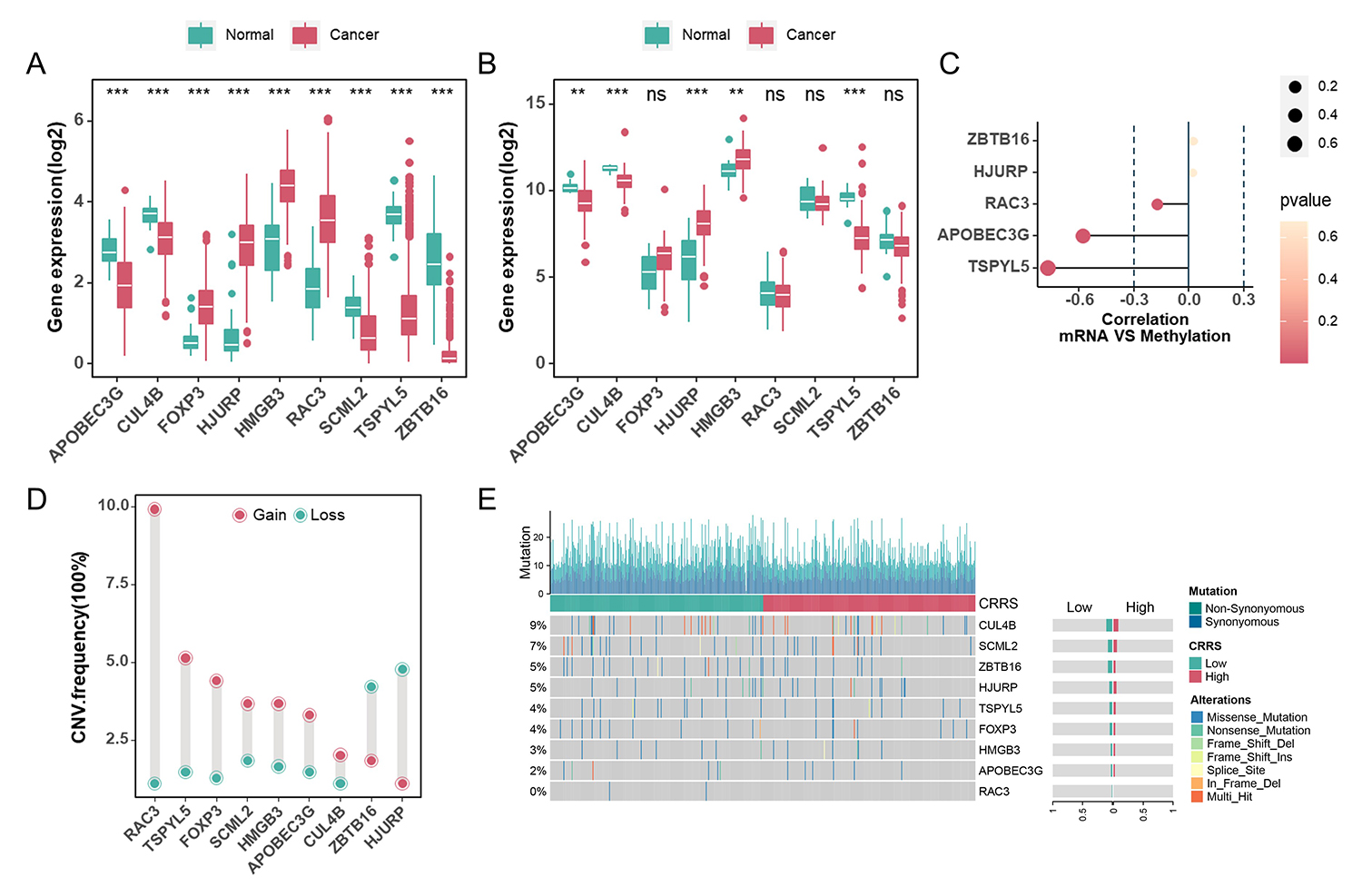

Supplement: Supplementary Figure 4 — The expression and genetic alterations of 9 TME-associated CRs in endometrial carcinoma. (A, B). The difference in mRNA expression levels of 9 TME-associated CRs between normal and endometrial carcinoma samples in TCGA-UCEC (A) and GSE17025 (B). (C). The correlation between expression levels and methylation of promoters of 9 TME-associated CRs. (D, E). The CNV frequency (D) and the mutation frequency (E) of 9 TME-associated CRs were prevalent. [file Image_4.jpeg]

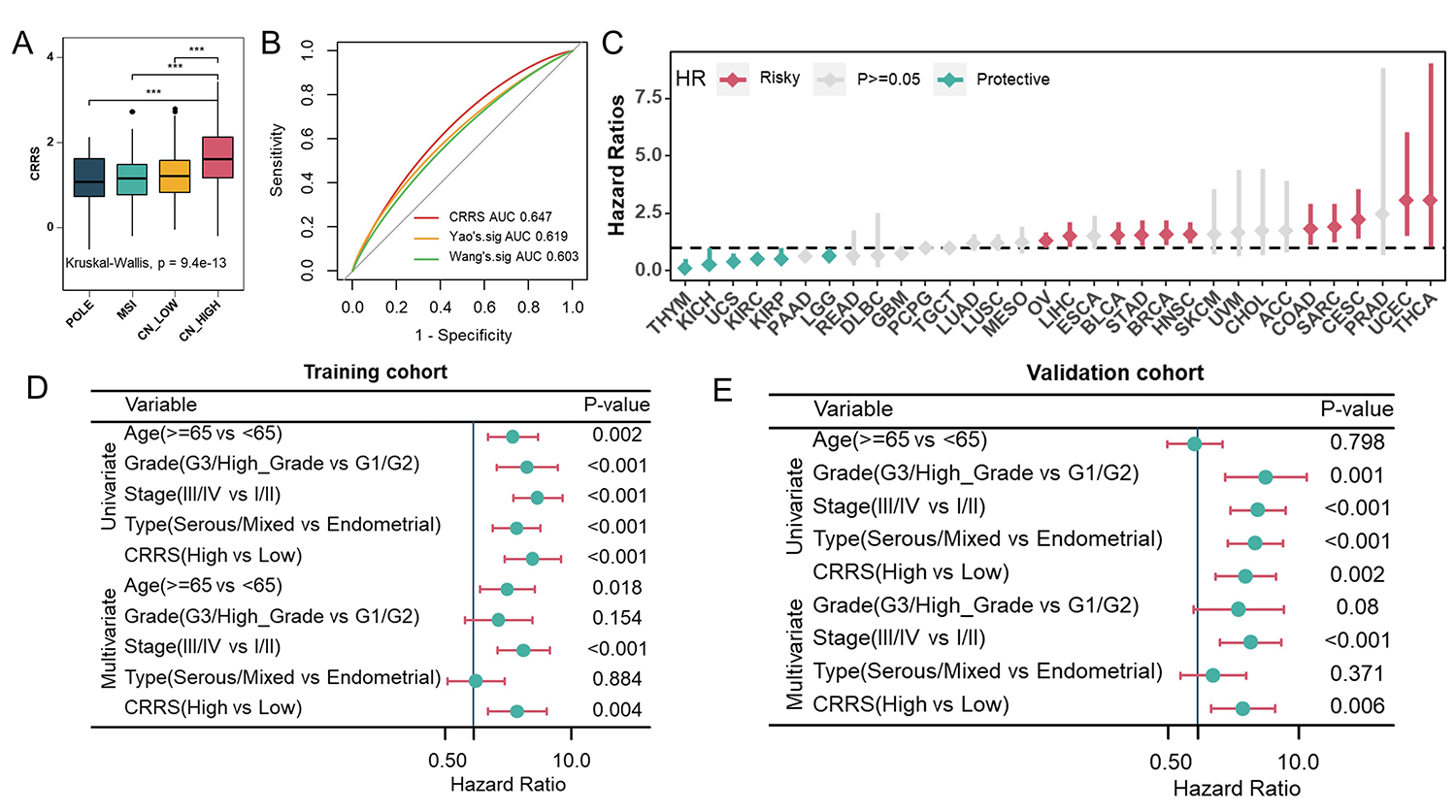

Supplement: Supplementary Figure 5 — Associations between CRRS and clinicopathological features. (A). The distribution of CRRS in TCGA-UCEC molecular subtypes in the entire cohort. (B). The AUC for CRRS and other prognostic signatures in the entire cohort. (C). Prognostic performance of the CRRS in the TCGA pan-cancer dataset. (D, E). Univariate and multivariate Cox regression analyses were performed in the training (D) and validation (E) cohorts to assess the independent predictive ability of CRRS and other clinicopathological features for OS. [file Image_5.jpeg]

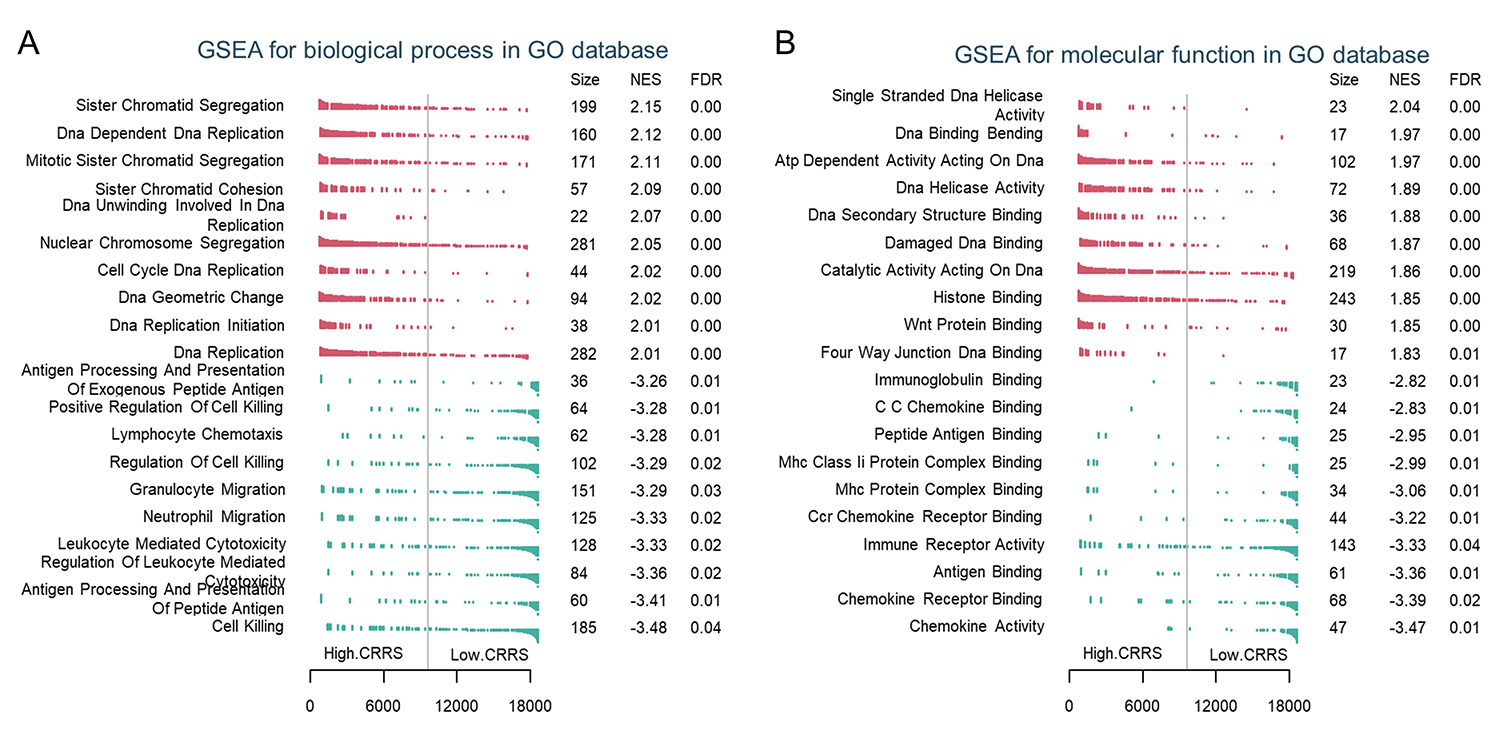

Supplement: Supplementary Figure 6 — The biological characteristics of different risk groups. (A). GSEA analysis of biological process gene sets from the GO dataset. (B). GSEA analysis of molecular function gene sets from the GO dataset. [file Image_6.jpeg]

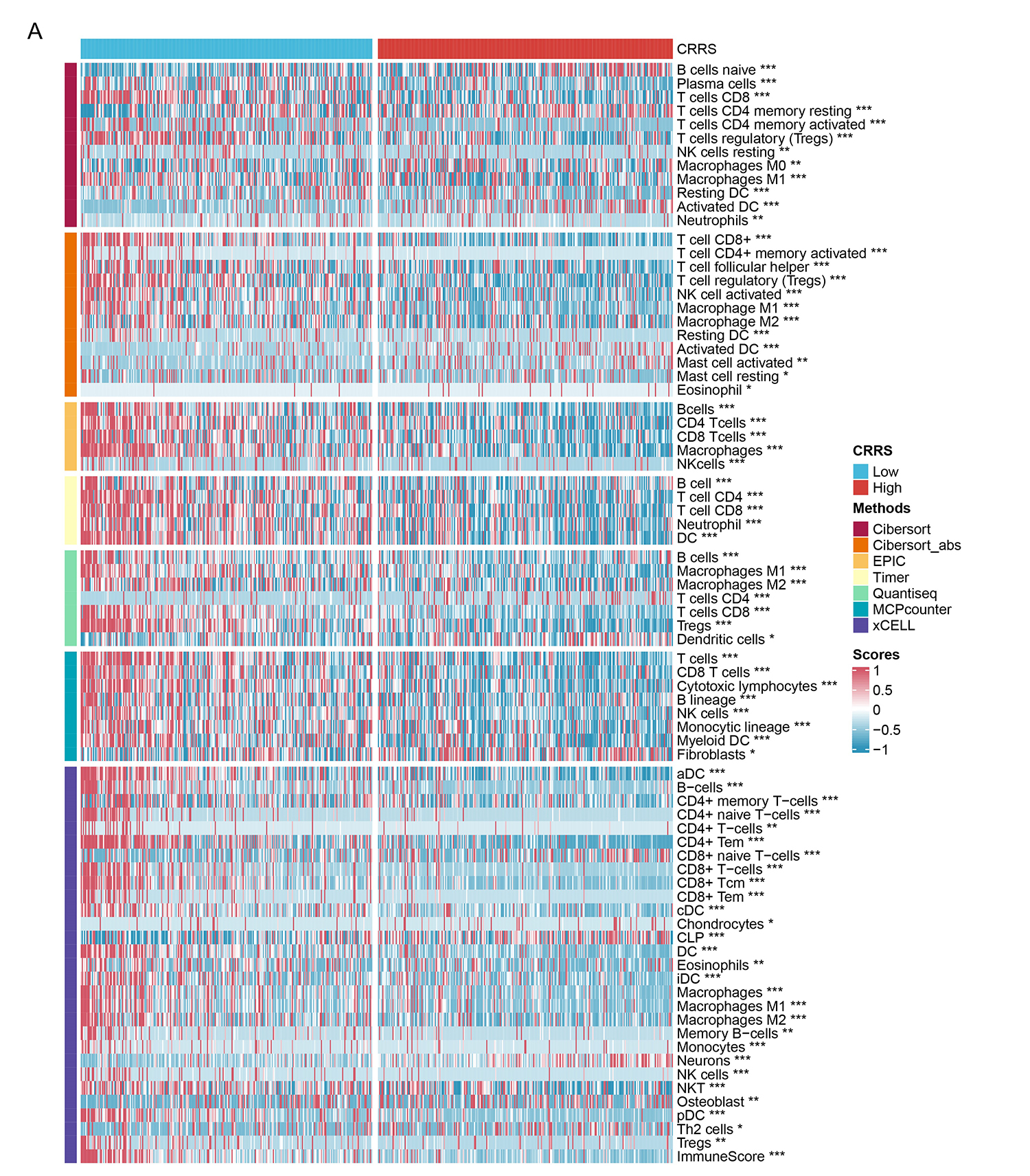

Supplement: Supplementary Figure 7 — Relationships between CRRS and the Tumor Microenvironment. (A). Relative cell abundance of CD8+ T cells, macrophages, DC cells, and NK cells were calculated by different algorithms in the low- and high-CRRS groups. [file Image_7.jpeg]

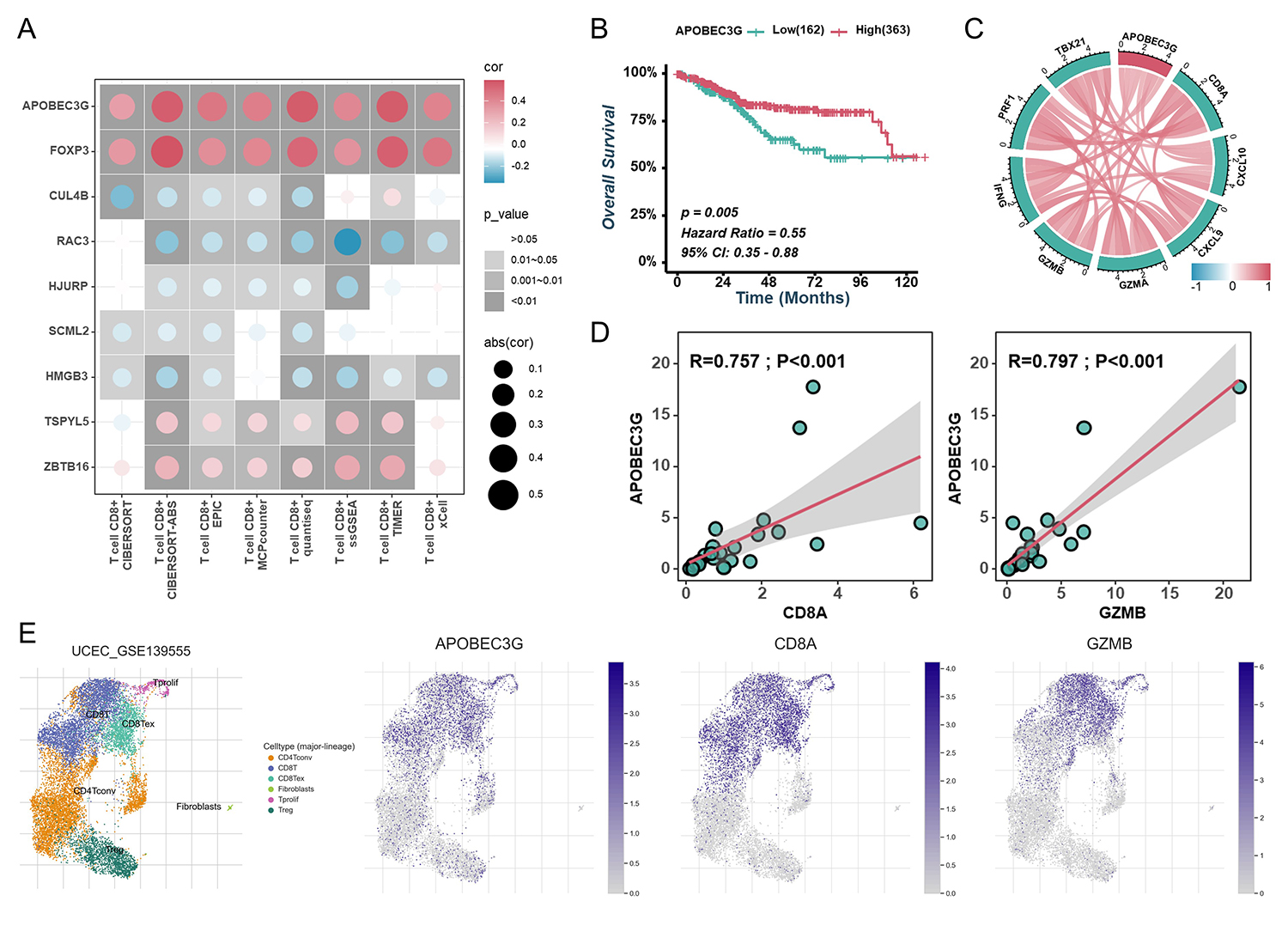

Supplement: Supplementary Figure 8 — APOBEC3G was positively correlated with CD8+ T cells in UCEC. (A). Multivariate analysis confirmed the correlation between the 9 TME-associated CRs and the levels of infiltration of CD8+ T cells. (B) Kaplan–Meier curve for OS between high- and low- APOBEC3G groups. (C) Correlation between the APOBEC3G and CD8+ T effector signature in TCGA-UCEC. (D) Validation of the correlation between APOBEC3G and CD8/GZMB by qRT-PCR. (E) The results of APOBEC3G, CD8, GZMB expression distribution in single cell dataset (UCEC-GSE139555). [file Image_8.jpeg]

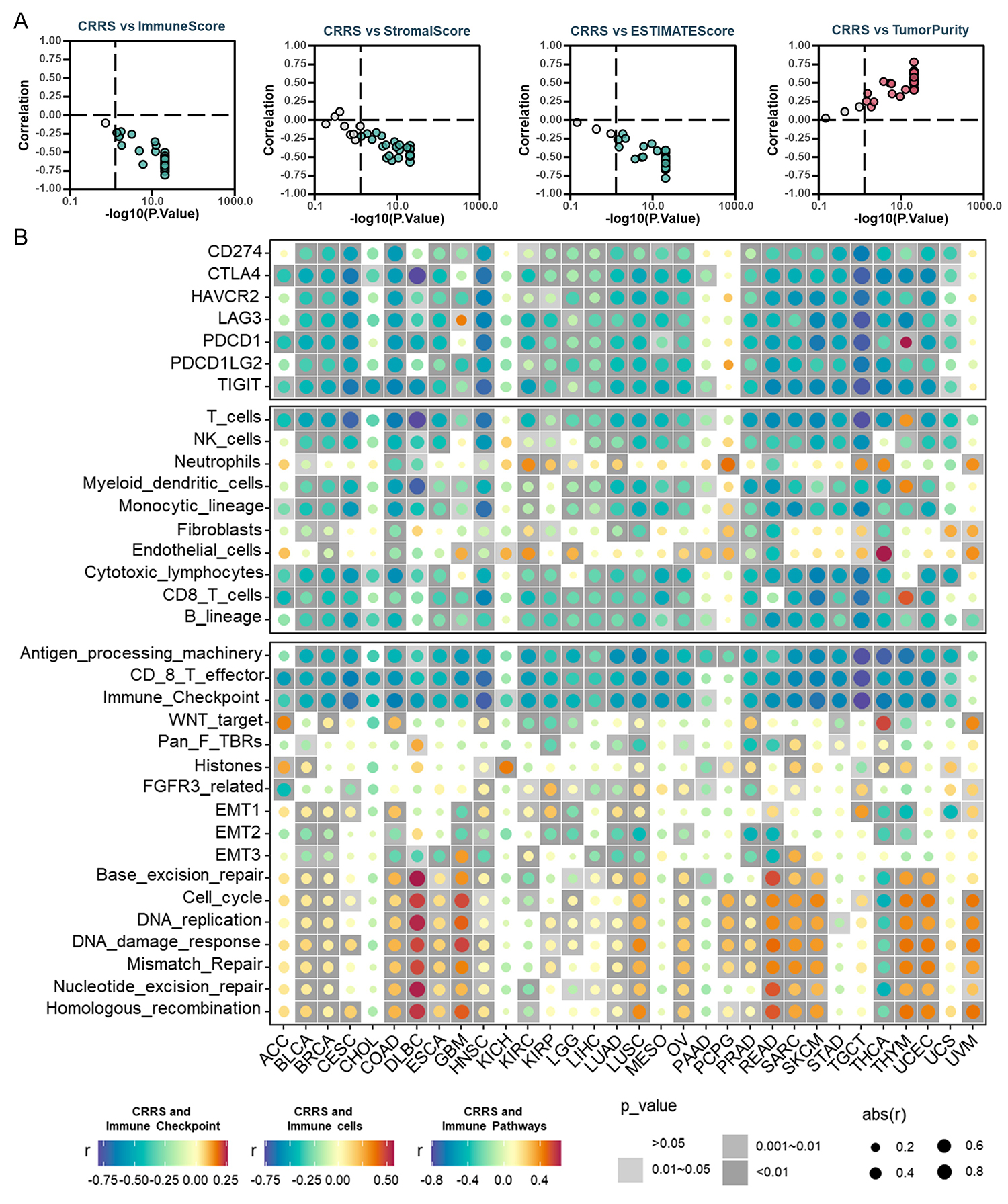

Supplement: Supplementary Figure 9 — CRRS and Tumor Microenvironment associations in the TCGA pan-cancer dataset. (A). The correlation between the CRRS and immune score, stromal score, ESTIMATE score, and tumor purity. (B). Heatmap illustrating the correlation between the CRRS and immune checkpoints, immune cells, and tumor microenvironment-related signatures. [file Image_9.jpeg]
